# Neuropathy, claw toes, intrinsic muscle volume, and plantar aponeurosis thickness in diabetic feet

**DOI:** 10.1186/s12891-020-03503-y

**Published:** 2020-07-23

**Authors:** Tadashi Kimura, Eric D. Thorhauer, Matthew W. Kindig, Jane B. Shofer, Bruce J. Sangeorzan, William R. Ledoux

**Affiliations:** 1 RR&D Center for Limb Loss and MoBility, VA Puget Sound Health Care System, Seattle, WA USA; 2 Departments of Orthopaedics and Sports Medicine, University of Washington, Seattle, WA USA; 3 Department of Orthopaedic Surgery, Jikei University School of Medicine, Tokyo, Japan; 4 Department of Mechanical Engineering, University of Washington, Seattle, WA USA

**Keywords:** Diabetic feet, Neuropathy, Claw toes, Intrinsic muscles, Plantar aponeurosis, Computed tomography

## Abstract

**Background:**

The objective of this study was to explore the relationships between claw toe deformity, peripheral neuropathy, intrinsic muscle volume, and plantar aponeurosis thickness using computed tomography (CT) images of diabetic feet in a cross-sectional analysis.

**Methods:**

Forty randomly-selected subjects with type 2 diabetes were selected for each of the following four groups (*n* = 10 per group): 1) peripheral neuropathy with claw toes, 2) peripheral neuropathy without claw toes, 3) non-neuropathic with claw toes, and 4) non-neuropathic without claw toes. The intrinsic muscles of the foot were segmented from processed CT images. Plantar aponeurosis thickness was measured in the reformatted sagittal plane at 20% of the distance from the most inferior point of the calcaneus to the most inferior point of the second metatarsal. Five measurement sites in the medial-lateral direction were utilized to fully characterize the plantar aponeurosis thickness. A linear mixed-effects analysis on the effects of peripheral neuropathy and claw toe deformity on plantar aponeurosis thickness and intrinsic muscle volume was performed.

**Results:**

Subjects with concurrent neuropathy and claw toes had thicker mean plantar aponeurosis (*p* < 0.006) and may have had less mean intrinsic muscle volume (*p* = 0.083) than the other 3 groups. The effects of neuropathy and claw toes on aponeurosis thickness were synergistic rather than additive. A similar pattern may exist for intrinsic muscle volume, but results were not as conclusive. A negative correlation was observed between plantar aponeurosis thickness and intrinsic muscle volume (R^2^ = 0.323, *p* < 0.001).

**Conclusions:**

Subjects with concurrent neuropathy and claw toe deformity were associated with the smallest intrinsic foot muscle volumes and the thickest plantar aponeuroses. Intrinsic muscle atrophy and plantar aponeurosis thickening may be related to the development of claw toes in the presence of neuropathy.

## Background

**Fig. 1:**
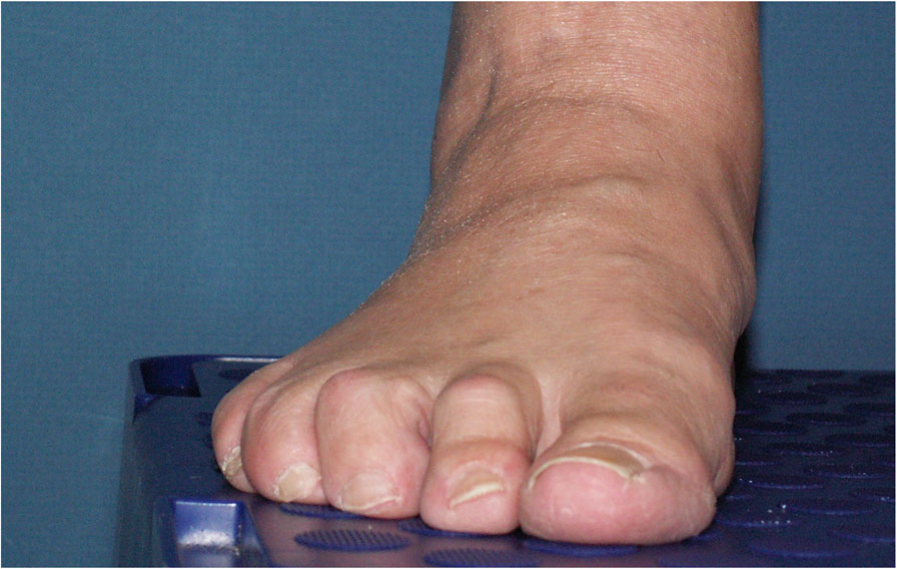
Representative claw toes. A claw toe (third toe) is defined as extended MTPJ, flexed proximal interphalangeal joint (PIPJ), and flexed distal interphalangeal joint (DIPJ), while a hammer toe (second toe) is defined as extended MTPJ, flexed PIPJ, and extended DIPJ. For this study, we will refer to both as “claw toes”

Diabetes currently affects more than 425 million people worldwide and is expected to surpass 629 million individuals by 2045 [1]. The rate of non-traumatic amputation has trended downward and is likely a result of improved preventive care, increases in revascularization interventions, and evolving orthopedic management [2]. However, there are still a significant number of diabetic amputations each year (108,000 in the United States in 2014 [3]) and it has been estimated that up to 80% of those are preceded by diabetic foot ulcers [4]. The development of diabetic foot ulcers is a multi-factorial process that has been associated with, among other factors, diabetic neuropathy, minor foot trauma, and foot deformities [5]. The most common deformity is at the metatarsophalangeal joints (MTPJ), which has been shown to have a prevalence as high as 85% in persons with a history of ulcers and amputation [6]. Characteristically, this forefoot deformity is commonly called a “claw toe” or a “hammer toe”. A claw toe is defined as extended MTPJ, flexed proximal interphalangeal joint (PIPJ), and flexed distal interphalangeal joint (DIPJ), while a hammer toe is defined as extended MTPJ, flexed PIPJ, and extended DIPJ. For this study, we will refer to both as “claw toes” (Fig. 1).

The literature is equivocal regarding the relationship between claw toes and diabetes. Initially, it was hypothesized that this deformity is caused by an imbalance between the extrinsic and intrinsic foot muscles [7]. When the intrinsic muscles are weakened, theoretically they are overpowered by the extrinsic muscles. This is exacerbated by dorsiflexion of the MTPJ, which causes the moment arm of the interosseous tendons to decrease, further reducing the ability of the intrinsic muscles to maintain plantarflexion of the proximal phalanx. However, some studies using magnetic resonance imaging (MRI) to quantify intrinsic muscle atrophy in diabetic neuropathic subjects have raised important questions on this issue, warranting further investigation [8–10]. Andersen et al. [8] and Bus et al. [9] found no relationship between claw toes and intrinsic atrophy, however, more recent work by Cheuy et al. did [10]. In addition to intrinsic muscle atrophy, there is also some evidence that the plantar aponeurosis (PA), claw toe deformity, and diabetes are closely linked [11–15]. The PA contributes to MTPJ stability by providing plantarflexion at this joint during weight bearing [16]. Several researchers have reported a relationship between claw toes and PA dysfunction [11, 12]. Others have found that subjects with diabetic feet have thicker PA than controls [13–15]. On the basis of these prior studies, the etiology of the claw toe deformity as it relates to diabetic foot complications is not well-understood. Many studies have evaluated the links between single factors, but the links between multiple factors remain unclear.

The purpose of this study was to explore the relationship between claw toes, neuropathy, intrinsic muscle volume, and plantar aponeurosis thickness using medical imaging. Understanding the etiology of claw toes is important in the management of diabetic foot complications via conservative treatment options and/or surgical reduction of this deformity. A better understanding of the role of intrinsic muscle atrophy in the development of claw toe deformity may inform treatment protocols for this deformity, such as strength training programs for the intrinsic muscles of the foot. This study also provides preliminary data on the expected magnitudes of differences in intrinsic muscle volume and PA thickness for those with and without claw toes. This should inform what kind of diagnostic tools (resolution) is required to track these changes.

## Methods

### Subjects

**Fig. 2:**
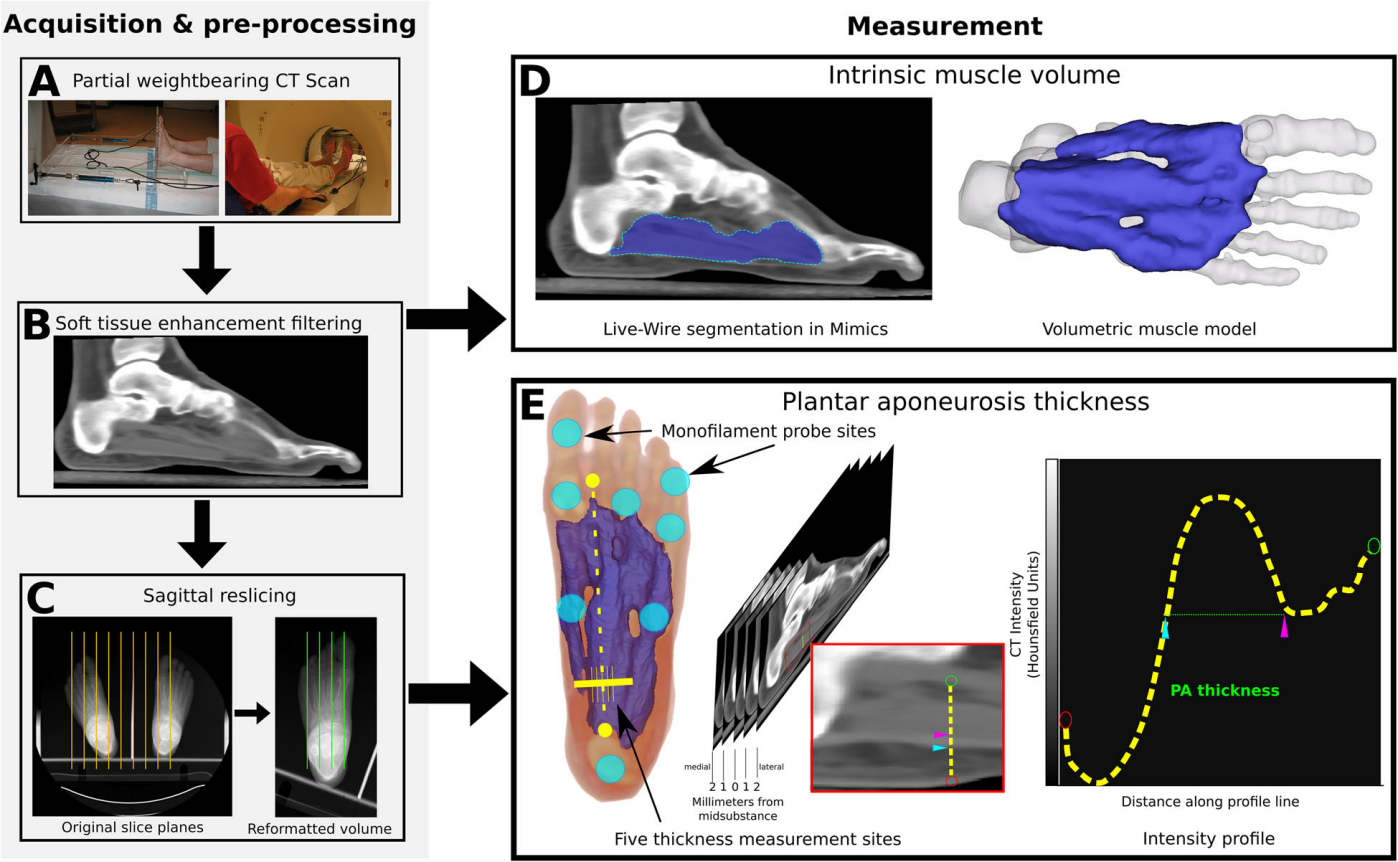
**a** Partial weight-bearing computed tomography (CT) loading frame. **b** Filtered CT image to reduce noise and enhance the contrast of the plantar tissues relative to the surrounding regions. **c** CT volumes were resliced using the multi-planar reconstruction tool to align the sagittal image plane with the most proximal point of calcaneus and the most distal point of second metatarsal head. **d** Final segmentation mask of intrinsic muscle of the foot. **e** Eight monofilament probe sites (cyan regions) were used to determine peripheral neuropathy and five measurement sites (yellow lines) in the medial-lateral direction were utilized to fully characterize the plantar aponeurosis (PA) thickness. Measuring the apparent thickness of the PA by using intensity profile measurements defined across the thickness of the PA at the measurement sites in the sagittal plane. Distance between the superior (pink)/inferior(blue) marks was taken as the PA thickness

This retrospective cross-sectional study was performed on a subset of subjects taken from a larger study of over 220 subjects with diabetes recruited from clinics at the Department of Veterans Affairs Puget Sound Health Care System, with approval from the Institutional Review Board. Written consent was obtained. Subjects were excluded for a variety of reasons including foot ulceration, bilateral foot amputation, non-ambulatory, inability to perform the protocol due to medical or psychiatric reasons, or inability to provide informed consent. Claw toe deformity was defined as an extended MTPJ, flexed PIPJ, and flexed DIPJ, and determined via clinical examination by a single rater. Peripheral neuropathy was defined as insensitivity to a 10-g Semmes-Weinstein monofilament at one of eight plantar locations (hallux, fifth toe, first metatarsal head, third metatarsal head, fifth metatarsal head, medial midfoot, lateral midfoot and heel) (Fig. 2e) [17, 18]. From this initial cohort, 10 subjects with type 2 diabetes were chosen to populate each of the following four groups: 1) peripheral neuropathy with claw toes (N+C+), 2) peripheral neuropathy without claw toes (N+C−), 3) non-neuropathic with claw toes (N−C+), and 4) non-neuropathic without claw toes (N−C−). Subject groups were balanced by confirming similar between-group distributions of sex, age, body mass index (BMI), duration of diabetes, and glycated hemoglobin (HbA1c). Ten subjects per group were chosen as a sample of convenience. Out of a possible 8 monofilament testing sites, neuropathic subjects had insensitivity in 5 ± 2.5 locations (mean ± standard deviation).

### CT images

Subjects underwent bilateral foot and ankle CT scanning (MX8000 IDT 16; Philips, the Netherlands; 140 kVp, 70 mA, 0.976 × 0.976 × 1.00 mm slices) in a custom-designed partial weight-bearing loading apparatus (target load = 20% body weight) in a neutral foot position [19] (Fig. 2a). CT volumes were imported into Mimics (version 21, Materialise, Leuven, Belgium). These CTs were originally acquired for a larger study focused on quantifying bone shape and position, and so the reconstruction kernel and imaging parameters were not optimized for soft tissue visualization. To approximate a soft tissue reconstruction kernel, the CT image data were filtered in Mimics to reduce noise and enhance the contrast of the plantar tissues relative to the surrounding regions. The following filter banks were used: one iteration of binomial blur, followed by 3 × 3 median filtering, followed by one iteration of binomial blur, followed by 5 × 5 median filtering. This sequence of filters was optimized via experimentation on a subset of our scan data and resulted in acceptable noise reduction in the image intensities without adversely compromising the delineation of the plantar tissue borders (Fig. 2b).

### Measurements

All measurements were blinded and performed by an orthopedic surgeon (TK) who has specialized in foot and ankle surgery for approximately 10 years.

### Plantar Aponeurosis thickness measurement

CT volumes were resliced by using Mimics’s multi-planar reconstruction tool to locate the most proximal point of the calcaneus and the most distal point of the head of the second metatarsal on axial images; the volume was then resliced in the sagittal plane containing these two points (Fig. 2c). PA thickness was measured in the resliced sagittal plane at 20% of the distance from the most inferior point of the calcaneus to the most inferior point of the second metatarsal as described by D’Ambrogi et al. [20]. Five measurement sites in the medial to lateral direction were utilized to appropriately characterize the PA thickness and reduce measurement error (the mid-substance of the PA central component, 1 mm medially, 2 mm medially, 1 mm laterally, 2 mm laterally). The PA tissue near the heel on the filtered sagittal CT appeared as a brighter region (higher Hounsfield units from greater radio-attenuation) relative to the surrounding tissues. To measure the apparent thickness of the PA, Mimics’s intensity profile tool was used to define measurements across the thickness of the PA at the measurement sites in the sagittal plane (Fig. 2e). The operator utilized the sagittal image data in conjunction with the intensity profile plots to demarcate the superior and inferior borders of the PA for each of the five measurement sites. All operations were performed while blinded to the foot group.

### Intrinsic muscle segmentation and volume measurement

The intrinsic muscles of the foot were initially segmented from the filtered images in Mimics using the LiveWire semi-automatic segmentation tool. The resulting segmentation masks were eroded by three pixels uniformly, and the Smart Expand segmentation tool was then used to grow the segmentations back away from the center until the computer detected a boundary in the images. Given the reduced noise in the filtered (low-pass) images, the Smart Expand tool (based on the Level-set segmentation method) should expand to fill the regions of the intrinsic muscles and stop at the tissue boundaries. Final muscle segmentation masks (Fig. 2d) were rendered as three-dimensional (3D) surface models of segmented bones and visually inspected for errors. The intrinsic muscle volume was calculated in Mimics as the volume of the segmentation mask based on the size and number of voxels that constitute the muscle mask. Foot length, defined as the distance from the most inferior point of the calcaneus to the most inferior point of the second metatarsal, was included as a covariate to account for foot size. All operations were performed in a similar manner irrespective of subject group, i.e., subject group was unknown during processing. Note that every attempt was made to determine borders between muscle and fat, but any fatty infiltration across the muscle, as seen in diabetic feet [9, 10, 16], would have incorrectly been considered muscle.

### Statistical analysis

The chi-squared test was used to verify equal distributions of sex between each group. One-way analysis of variance (ANOVA) was used to verify differences in age, BMI, foot length, duration of diabetes and HbA1c levels. Repeatability and reliability of the muscle volume and PA thickness measurements were assessed using intraobserver and interobserver correlation coefficients (ICC). To assess the reliability of intrinsic muscle volume measurements, five subjects, including one from each group, were segmented a second time, 2 weeks later, by the same surgeon (TK). The rater was blinded to the first segmentation and the presentation order was randomized to prevent memory bias. To assess the reliability of the PA thickness measurements, measurements were performed by two biomechanical engineers with at least 5 years of experience in processing medical images of the foot and ankle (MK and WR) in addition to the measurements by TK. PA thickness measurements were repeated three separate times on 12 randomly selected subjects after intervals of at least 5 days.

Linear regression was used to assess the association between intrinsic muscle volume (the dependent variable) and presence of neuropathy and claw toe deformity. Linear mixed effects regression [21] was used to assess the association between PA thickness (the dependent variable) and presence of neuropathy and claw toe deformity with study participant as a random effect. For both models, presence of neuropathy, claw toe deformity, and presence of neuropathy by claw toe deformity interaction were fixed effects. The model of intrinsic muscle thickness also included foot length as a fixed effect covariate. Post hoc pair-wise comparisons among the four subgroups (presence of claw toe deformity (C+) and presence of neuropathy (N+), or N+C+, N+C−, N−C+, N−C−) were performed. The significance of pair-wise differences was tested using the single-step simultaneous inference method [22] to adjust for the inflation of the Type 1 error due to performing seven sets of pairwise comparisons. Finally, linear regression was used to assess the correlation between mean participant PA thickness (the dependent variable) and intrinsic muscle thickness with foot length as a covariate. Analyses were performed using R statistical software [23]. Type 1 error was set at 0.05.

## Results

Subject demographic characteristics demonstrated that sex distribution (*p* = 0.50), age (*p* = 0.54), BMI (*p* = 0.46), foot length (*p* = 0.26), duration of diabetes (*p* = 0.21), and HbA1c level (*p* = 0.39) were not statistically different between each group (Table 1). As a result, these non-significant variables were excluded from the linear mixed-effects model. The mean ± standard deviation [maximum, minimum] PA thickness across all subjects was 3.68 ± 1.30 mm [1.91, 8.10]. The mean ± standard deviation [minimum, maximum] intrinsic muscle volume across all subjects was 99.53 ± 31.13 cm^3^ [26.43, 179.51].

**Table 1:** Patient characteristics

	Peripheral neuropathy		Non-neuropathic		*p*-value
	Claw toes (N+C+)	No claw toes (N+C-)	Claw toes (N-C+)	No claw toes (N-C-)	
**Number of subjects**	10	10	10	10	
**Sex (male/female)**	9/1	9/1	7/3	9/1	0.5
**Age (years)**	60.2 ± 6.0	57.2 ± 5.8	59.9 ± 10.3	56.3 ± 5.7	0.54
**Body mass index (kg/m**^**2**^**)**	37.6 ± 4.6	34.9 ± 8.3	34.5 ± 4.5	33.9 ± 3.5	0.46
**Foot length (mm)**	162.2 ± 11.9	158.4 ± 8.1	155.7 ± 11.4	152.7 ± 11.5	0.26
**Diabetes duration (years)**	9.3 ± 3.8	7.2 ± 5.3	8.0 ± 6.0	4.8 ± 3.6	0.21
**HbA**_**1C**_**(%)**	6.7 ± 0.7	7.7 ± 0.8	7.8 ± 0.6	7.3 ± 2.0	0.39

Intrinsic muscle volume segmentations were highly repeatable (ICC = 0.99, 95% confidence interval [CI] range: 0.95–0.99). The difference in total intrinsic muscle volume between the first and second segmentations ranged from − 4.9 to + 4.2%. The inter-class correlation coefficient of all PA thickness measurements between the three raters was ICC(A,1) = 0.84 (95% CI range: 0.78–0.89), and the intra-class correlation coefficients of the three raters were ICC_rater1_ = 0.98, ICC_rater2_ = 0.92, and ICC_rater3_ = 0.77. Rater 1 (TK) exhibited the highest repeatability amongst raters and subsequently performed all the PA thickness measurements for the study data analysis (95% CI range: 0.97–0.99).

**Fig. 3:**
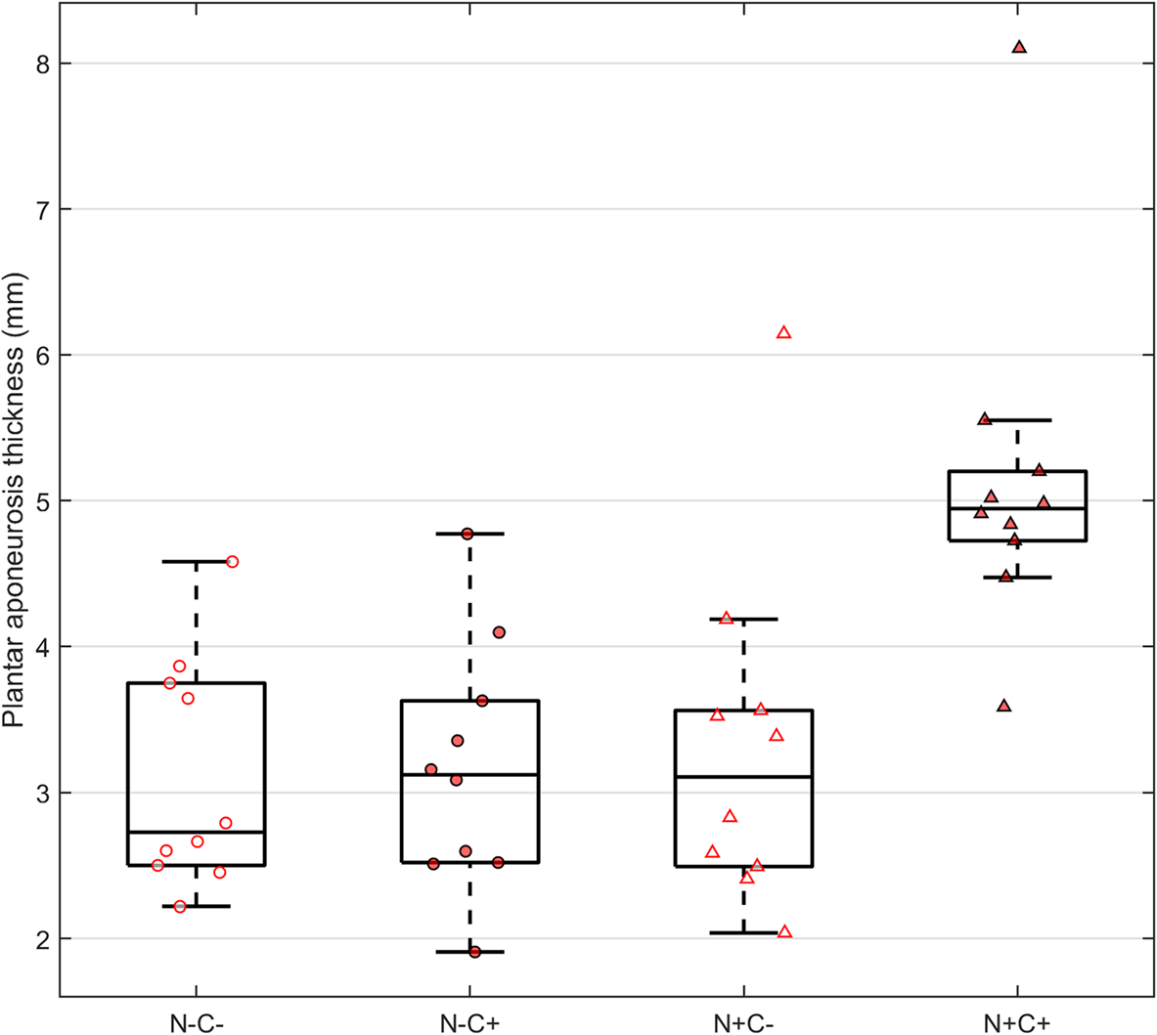
Mean plantar aponeurosis (PA) thickness by subgroup

For PA thickness, there was a significant interaction (*p* < 0.006) between neuropathy and claw toe status, as subjects with both factors had increased PA thickness (Table 2, Fig. 3). The effects of neuropathy and claw toes on plantar aponeurosis thickness was synergistic rather than additive. Subjects with both neuropathy and claw toe deformity (N+C+) had thicker PA compared with: neuropathic non-claw toe (N+C−, mean difference = 1.82 mm, 95% CI: [0.60, 3.05], *p* = 0.002), non-neuropathic, claw toe subjects (N−C+, mean difference = 1.98 mm, 95% CI: [0.76, 3.20], *p* < 0.001), and non-neuropathic, non-claw toe subjects (N−C−, mean difference = 2.03 mm, 95% CI: [0.81, 3.25], *p* < 0.001).

**Table 2:** Estimated marginal means, standard error, and range of intrinsic muscle volume and plantar aponeurosis thickness by subgroup from their respective linear models

	Neuropathy	Claw toe	Estimated mean	Standard error	Range
**Plantar aponeurosis thickness (mm)**	N+	C+	5.14	0.321	0.58
	N+	C−	3.31	0.321	0.59
	N−	C+	3.16	0.321	0.59
	N−	C−	3.11	0.321	0.59
**Intrinsic muscle volume (cm**^**3**^**)**	N+	C+	69.30	8.87	36.00
	N+	C−	109.80	8.63	35.00
	N−	C+	104.60	8.64	35.10
	N−	C−	114.40	8.83	35.80

**Fig. 4:**
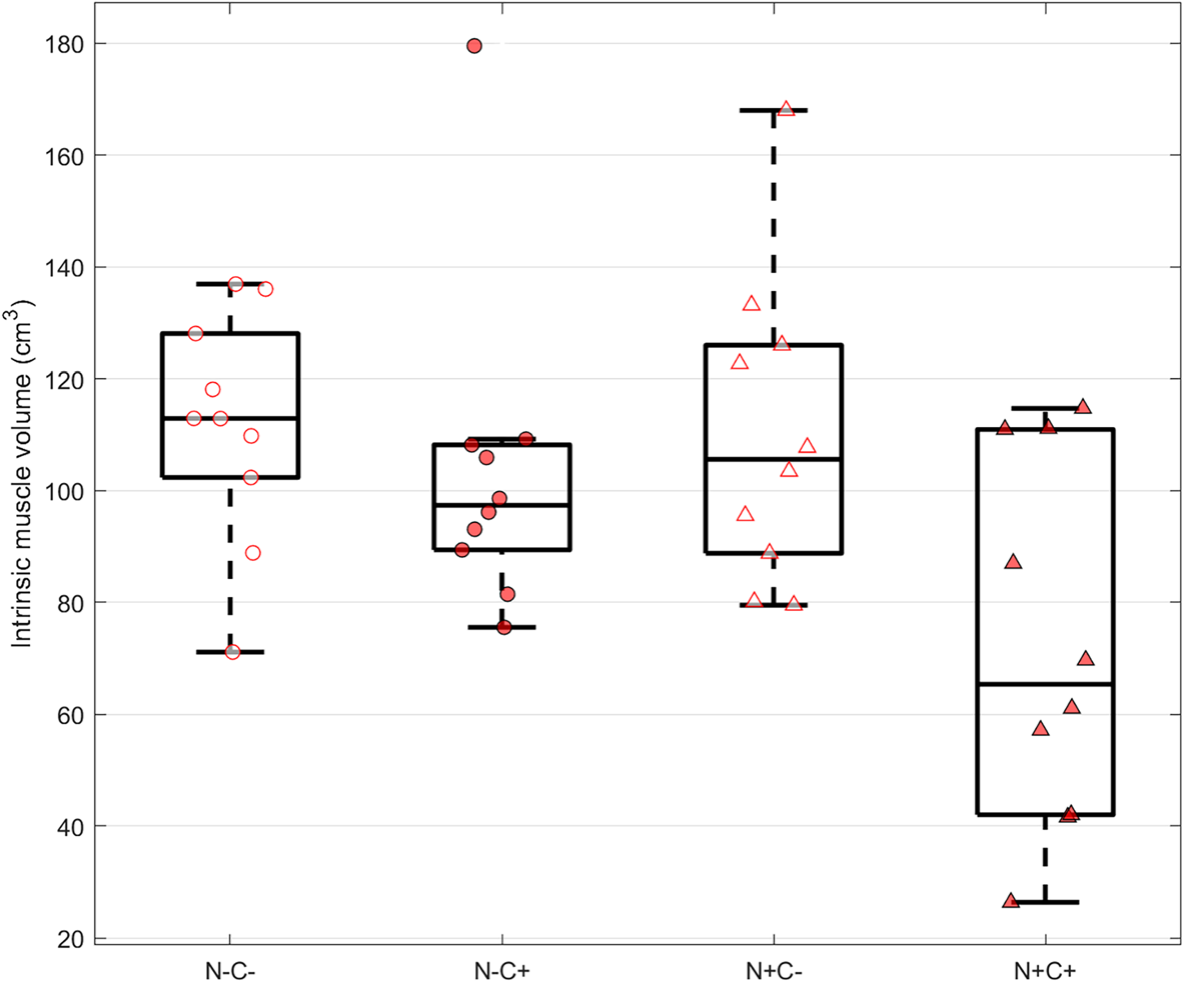
Mean intrinsic muscle volume (IMV) by subgroup

**Fig. 5:**
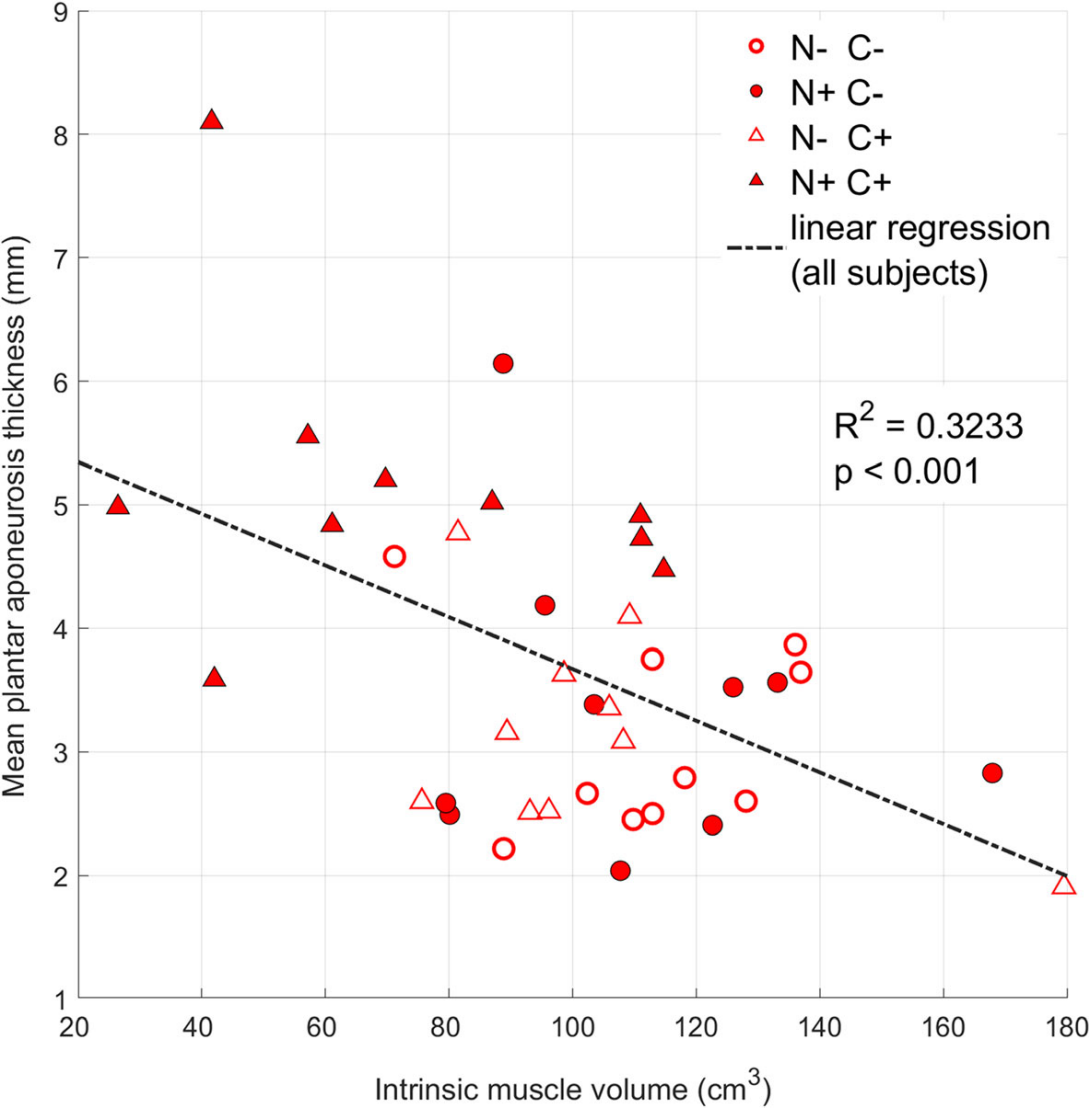
Correlation between plantar aponeurosis thickness and normalized intrinsic muscle volume

For intrinsic muscle volume, there was borderline interaction (*p* = 0.083) between neuropathy and claw toe status, as subjects with both factors has decreased intrinsic muscle volume (Table 2, Fig. 4). Subjects with both neuropathy and claw toe deformity (N+C+) had smaller intrinsic muscle volumes relative to: neuropathic non-claw toe subjects (N+C−, mean difference = 40.52 cm^3^, 95% CI: [7.36, 73.70], *p* = 0.012), non-neuropathic, claw toe subjects (N−C+, mean difference = 35.31 cm^3^, 95% CI: [1.63, 69.00], *p* = 0.037), and non-neuropathic, non-claw toe subjects (N−C−, mean difference = 45.10 cm^3^, 95% CI: [10.51, 79.70], *p* = 0.007). All contrasts between other groups (N+C−, N−C+, N−C−) were not significant. When the marginal means were examined further we found that presence of neuropathy was significantly associated with lower mean intrinsic muscle volume only for those with claw toes (N+ minus N− for C+: − 35.3 cm^3^, 95%CI [− 69, − 2], *p* = 0.037; C−: − 4.6 cm^3^ 95%CI [− 38, 29], *p* = 0.98), and presence of claw toes was significantly associated with lower intrinsic muscle volume only for those with neuropathy (C+ minus C− for N+: − 40.5 cm^3^, 95%CI [− 74, − 7], *p* = 0.012; N−: − 9.8 cm^3^, 95%CI [− 43, 23], *p* = 0.85). Whether the effect of neuropathy and claw toes in IMV was additive or synergistic could not be clearly determined from this data. A negative correlation was seen between PA thickness and intrinsic muscle volume (*R*^2^ = 0.323, *p* < 0.001) (Fig. 5).

## Discussion

Previous studies have shown that multiple factors have been associated with claw toe deformity, but the relationships between each of these factors remain unclear. The main goal of this study was to explore the association between claw toes, peripheral neuropathy, intrinsic muscle volume, and PA thickness.

Our study also found that for PA thickness, neuropathy and claw toe status significantly interacted (*p* < 0.006); the N+C+ group was significantly thicker than every other group. Several studies have reported thicker PA in diabetic feet than in control feet [13–15, 20], and Duffin et al. concluded that the pathogenesis of PA thickening may have been caused by non-enzymatic glycation and mechanical loading [14]. However, one of these studies [13] compared only diabetic neuropathic subjects to controls and another [14] examined only young subjects without mention of neuropathy. The other two studies [15, 20] examined controls, diabetic subjects, diabetic neuropathic subjects, and diabetic neuropathic subjects with recently healed ulcers; neither found significant differences in PA thickness between the diabetic and the diabetic neuropathic groups. In other words, like our study, there was no evidence that isolated neuropathy was associated with increased PA thickness. Moreover, none of these studies that measure the effect of diabetes on PA thickness also considered claw toes. Our results indicated that diabetic neuropathic feet with claw toes had thicker PA than other groups, but we are unaware of a similar study comparing PA thickness between feet with or without claw toe deformity and with or without neuropathy. Although this would need to be confirmed on a larger, and likely prospective study, the implication is that more pathological feet (those with concurrent neuropathy and claw toes) have both a thicker PA, and, as evidenced in this study (Fig. 5), less intrinsic muscle.

Our findings indicate that neuropathic feet with claw toes have decreased intrinsic muscle; it is likely the effect of neuropathy and claw toes is synergistic, as suggested by our borderline non-significant interaction term (*p* = 0.083). Of note, the N+C+ group had the smallest intrinsic muscle volume among all groups. Reduction of intrinsic muscle volume in the diabetic neuropathic foot has been demonstrated previously [8, 9]. Cheuy et al. [10] and Robertson et al. [24] also found a relationship between intrinsic muscle volume and forefoot deformity. They reported that reduced lean muscle volume or muscle density was associated with increased hyperextension at the MTPJ in diabetic patients with neuropathy. However, conflicting results regarding the relationship between intrinsic muscle volume reduction and claw toes have been shown in other literature. Andersen et al. [8] and Bus et al. [9, 16] reported no relationship between muscle volume and MTPJ deformity. Bus et al. found a 73% decrease in intrinsic muscle cross sectional area between patients with diabetic neuropathy and non-neuropathic controls, but only two of eight patients with neuropathy had toe deformities [9]. Anderson et al. found that patients with diabetic neuropathy had an intrinsic muscle volume just over half that of either controls or patients with no diabetic neuropathy, but none of the diabetic patients with neuropathy had toe deformities [8]. The reasons for these differences are likely due to variations in experimental techniques or study aims. For example, Andersen et al. was studying diabetic neuropathic feet and retrospectively considered the presence of claw toes [8]. Further, Bus et al. did not explicitly enroll claw toe subjects and used a single CT slice to estimate intrinsic muscle volume [9, 16]. Both studies also used nerve conduction velocities to confirm peripheral neuropathy, which our study did not. In contrast, Cheuy et al. reported that less forefoot lean muscle tissue was associated with greater MTPJ deformity [10]. This group, whose results agree with the current study, truly measured the 3D intrinsic muscle volume as we did. Even though image segmentation and 3D analysis of the muscle volume is time-consuming, thus far this methodology is showing relationships not elucidated by the two-dimensional methods.

We also showed that there was a negative correlation between muscle volume and PA thickness. Greenman et al. found a reduction in the thickness of the intrinsic foot muscle at the level of the metatarsal joint in both neuropathic and non-neuropathic patients, and these findings suggest that intrinsic foot muscle atrophy can occur in diabetic feet even before neuropathy develops [25]. This atrophy of intrinsic muscle may be concurrent with the non-enzymatic glycation of the PA that also occurs in early-stage diabetes. As the atrophy progresses, the mechanical load experienced by the PA during walking or weightbearing activity increases. It was inferred that the further progress of PA thickening, along with the decreased intrinsic muscle volume, may both lead to claw toe deformity. However, given the lack of longitudinal data, we cannot conclude whether intrinsic muscle atrophy or the PA thickening presents first, or they develop concurrently.

It should be noted that in our study, the mean PA thickness across all subjects was 3.68 ± 1.34 mm, which is similar to the average value 4.2 ± 0.9 mm reported by the measurements of Boulton et al. using CT images [13] as we did. However, using ultrasound, Duffin et al., Giacomozzi et al., and D’Ambrogi et al. measured diabetic PA thickness that was less than our results: 1.6 ± 1.8mm (interquartile range), 2.9 ± 1.2 mm, respectively [14, 15, 20]. This difference is likely due to differences in imaging modality, i.e., CT vs. ultrasound, and the fact the Duffin et al. measured PA thickness beneath the first metatarsal head.

One potential clinical implication is that while patient education and adequate control of blood glucose level are certainly important, our results indicate that a strength training program for the intrinsic muscles of the foot [26, 27] may help to improve specific muscle strength and prevent muscle atrophy. This can minimize the risk of development of deformity that could lead to amputation.

There were several potential limitations to this study. First, the duration and the severity of neuropathy and claw toes was not determined. Second, we did not account for the possibility that the deformity might have been caused by ill-fitting footwear and congenital anomalies, especially for patients with claw toes without neuropathy. Third, we did not consider or quantify variation in patient daily activity levels (e.g., step counts), which may be related to a variety of health factors. Fourth, as this is secondary analysis, we did not perform an a priori power analysis and it is certainly possible that our study is underpowered. A larger sample size certainly would have reduced the variability. Fifth, the threshold for diagnosing neuropathy is controversial. Wang et al. concluded in a meta-analysis review that monofilament tests have limited sensitivity for screening diabetic peripheral neuropathy [28]. Feng et al. suggests testing at three sites including the plantar aspects of the great toe, the third metatarsal, and the fifth metatarsal, to maximize the diagnostic value of Semmes Weinstein monofilament examination [29]. In our current study, we performed monofilament testing at eight plantar locations, including the three recommended sites by Feng et al. As such, we are confident in our classifications of neuropathic patients, despite the limitations of monofilament assessment described by Wang. However, it is possible that using a non-binary classification approach, such as fuzzy logic, to diagnose diabetic neuropathy severity [30, 31] might have been more descriptive. Additionally, we did not quantify the function and strength of the intrinsic muscles and PA, and the measured parameters (muscle volume and PA thickness) do not necessarily reflect these factors. The CT scanning and image analysis presented multiple challenges. We had seven subjects in which there was incomplete contact between the heel and the foot plate on CT images (i.e., the subjects were not weight-bearing on their heels). We defined the profile line for measuring PA thickness as the “vertical” in the image coordinate system (see Fig. 2e). This was considered a potential source of bias in measurements of PA thickness because some of the subjects’ feet were tilted with respect to this vertical axis. The maximum angle error was approximately 10 degrees. However, even in the worst-case scenario of no heel contact, our values were within 2% of the “true PA thickness”. This equates to about 0.07 mm of error for the average 3.68 mm-thick PA tissue. A final limitation of using CT to assess intrinsic muscle size is that CT is generally not the modality of choice for these kinds of soft tissue measurements. Rather, this paper is a secondary analysis of an osseous foot structure study and, as such, the CT technique was not optimized for soft tissue. Finally, fatty infiltration, as occurs in diabetic feet, was not directly accounted for in our methods and, as such, fatty tissue might have incorrectly been considered as intrinsic muscle.

## Conclusion

Subjects with concurrent neuropathy and claw toes had thicker mean plantar aponeurosis and may have had less mean intrinsic muscle volume. The effects of neuropathy and claw toes on aponeurosis thickness were synergistic rather than additive. A similar pattern may exist for intrinsic muscle volume, but results were not as conclusive. We suggest that intrinsic muscle atrophy, increased PA thickness, and neuropathy are all potentially related to the complex pathomechanics of the development of claw toe deformity. It may be helpful for patients with diabetes to have an active intervention to prevent intrinsic muscle atrophy and PA thickening or to be screened for these changes prior to the development of claw toes.

## Data Availability

The datasets used and/or analyzed during the current study are available from the corresponding author on reasonable request.

## Abbreviations

3D: Three dimensional
ANOVA: One-way analysis of variance
BMI: Body mass index
CI: Confidence interval
C+: Claw toe deformity
CT: Computed tomography
DIPJ: Distal interphalangeal joint
HbA1c: Glycated hemoglobin
ICC: Intra-observer and inter-observer correlation coefficients
MRI: Magnetic resonance imaging
MTPJ: Metatarsophalangeal joints
N+: Presence of neuropathy
N+C+: Peripheral neuropathy with claw toes
N+C−: Peripheral neuropathy without claw toes
N−C+: Non-neuropathic with claw toes
N−C−: Non-neuropathic without claw toes
PA: Plantar aponeurosis
PIPJ: Proximal interphalangeal joint
